# Incidence and spatial distribution of cases of dengue, from 2010 to 2019: an ecological study

**DOI:** 10.1590/1516-3180.2020.0111.R1.24092020

**Published:** 2020-12-14

**Authors:** Petrúcio Luiz Lins de Morais, Priscila Mayrelle Silva Castanha, Ulisses Ramos Montarroyos

**Affiliations:** I BSc. Assistant Professor, Department of Biological Sciences, Universidade de Pernambuco (UPE), Garanhuns (PE), Brazil.; II MSc, PhD. Research Collaborator, School of Medical Sciences, Institute of Biological Sciences, Universidade de Pernambuco (UPE), Recife (PE), Brazil.; III BSc. Adjunct Professor, School of Medical Sciences, Institute of Biological Sciences, Universidade de Pernambuco (UPE), Recife (PE), Brazil.

**Keywords:** Environmental health, Arbovirus infection, Severe dengue, Dengue virus, Epidemiology, Frequency, Surveillance, Spatial distribution

## Abstract

**BACKGROUND:**

Dengue is an arbovirus that has caused serious problem in Brazil, putting the public health system under severe stress. Understanding its incidence and spatial distribution is essential for disease control and prevention.

**OBJECTIVE:**

To perform an analysis on dengue incidence and spatial distribution in a medium-sized, cool-climate and high-altitude city.

**DESIGN AND SETTING:**

Ecological study carried out in a public institution in the city of Garanhuns, Pernambuco, Brazil.

**METHODS:**

Secondary data provided by specific agencies in each area were used for spatial analysis and elaboration of kernel maps, incidence calculations, correlations and percentages of dengue occurrence. The Geocentric Reference System for the Americas (Sistema de Referência Geocêntrico para as Américas, SIRGAS), 2000, was the software of choice.

**RESULTS:**

The incidence rates were calculated per 100,000 inhabitants. Between 2010 and 2019, there were 6,504 cases and the incidence was 474.92. From 2010 to 2014, the incidence was 161.46 for a total of 1,069 cases. The highest incidence occurred in the period from 2015 to 2019: out of a total of 5,435 cases, the incidence was 748.65, representing an increase of 485.97%. Population density and the interaction between two climatic factors, i.e. atypical temperature above 31 °C and relative humidity above 31.4%, contributed to the peak incidence of dengue, although these variables were not statistically significant (P > 0.05).

**CONCLUSION:**

The dengue incidence levels and spatial distribution reflected virus and vector adjustment to the local climate. However, there was no correlation between climatic factors and occurrences of dengue in this city.

## INTRODUCTION

In the second half of the twentieth century, dengue fever spread throughout the tropics, threatening one-third of the world’s population. It caused feverish illness in around 50 to 100 million people, with records of 500,000 cases of severe illness.^1^ Dengue is caused by an arbovirus that is transmitted by the mosquitos *Aedes aegypti* and *Aedes albopictus*. Its symptoms range from an acute fever to a hemorrhagic condition, and can be caused by four different virus serotypes.^2^ Once an *Aedes* female has become infected, it can transmit the virus to humans through blood transfers for the rest of its life, which leads to greater potential for spreading the disease.^3^ *Aedes aegypti* also transmits other high-impact arboviruses such as chikungunya.^4^

Circulation of different virus serotypes has increased the number of infected patients, especially with the severe form of the disease.^5^ There is no specific therapy for dengue infections and supportive treatment can save lives.^6^

The first cases of dengue in Brazil were recorded in the state of Roraima, in the northwestern area of the Amazon region, in 1981.^7^

Dengue has become a serious public health problem in the city of Garanhuns, state of Pernambuco, northeastern Brazil. Over the last five years, the incidence of dengue has increased by 485.97%. Epidemiological studies have confirmed that the dengue virus, which first appeared in this region in 1986, presents high intensity of transmission. This research has therefore characterized a situation of lack of knowledge about the behavior of the virus and its vector.^8^

In Pernambuco, the dengue virus serotype three (DENV-3) was associated with the most severe symptoms of the epidemic, from 1995 to 2006.^9^ A total of 9,135 dengue cases were recorded in Pernambuco in 2018, corresponding to an incidence rate of 96.2 cases per 100,000 inhabitants. There were 31,056 dengue cases in 2019, with an incidence rate of 327.0 cases per 100,000 inhabitants, which was a percentage increase of 240.0%.

In the adjacent states of Alagoas and Paraiba, totals of 17,486 and 13,959 cases respectively were recorded in 2019, corresponding to incidence rates of 526.2 and 349.3 cases per 100,000 inhabitants.^10^ Throughout Brazil, a total of 1,439,471 probable dengue cases were recorded in the same year.^10^

Also in the same year, 2,384,029 cases of dengue were recorded throughout the Americas, corresponding to an incidence rate of 244.1 cases per 100,000 inhabitants, and 949 deaths were registered.^11^

## OBJECTIVE

To describe the incidence and spatial distribution of dengue cases in a medium-sized city with a seasonal climate and high altitude, located in Brazil’s northeastern region. Over the period studied (2010-2019), outbreaks and epidemics were observed, with an increased in the incidence rate of 485.97% over the last five years of that period.

## METHODS

### Study location

This descriptive ecological study was conducted in the city of Garanhuns, state of Pernambuco, northeastern Brazil, covering the years 2010 to 2019. Garanhuns is located 230 kilometers (km) west of the state capital, at an altitude of 896 meters (m) above sea level. It has an area of 472.5 square kilometers (km^2^) and an estimated population density in 2019 of 295.84 inhabitants per km^2^. The minimum temperature ranges from 15 to 16 degrees centigrade (°C) and the maximum, from 28 to 31.5 °C. The average rainfall over the study period was 68.90 mm per annum and the average relative humidity was 40.1%.^12^^,^^13^ The climate of Garanhuns is influenced by meteorological systems that cause rainfall mostly in March, June and July.^12^ The 2010 census registered a population of 129,408 inhabitants in this municipality, and a total of 139,788 inhabitants was estimated for 2019.^14^

### Data collection

Information about dengue cases was obtained from the following secondary data sources: the Brazilian Ministry of Health’s Notifiable Disease Information System (Sistema de Informação de Agravos de Notificação, Ministério da Saúde, SINAN/MS);^15^ the Pernambuco State Health Department; and the City of Garanhuns Health Department. Socioeconomic and demographic information was obtained from the 2010 census data held by the Brazilian Institute for Geography and Statistics (Instituto Brasileiro de Geografia e Estatística, IBGE).^14^ Climate data was collected from the Water and Climate Agency of the State of Pernambuco (Agência Pernambucana de Águas e Clima, APAC).^12^

### Data processing

The information contained in the SINAN database was used for georeferencing of dengue cases in the city. This was then incorporated into the Geographic Information System (GIS), within the Quantum GIS (QGIS) software (Open Source Geospatial Foundation, Chicago, USA), version 2.18.11, through which data from multiple sources were integrated. TerraView 4.4.2, from the Image Processing Division, National Space Research Institute (Instituto Nacional de Pesquisas Espaciais, INPE; São José dos Campos, SP, Brazil), was the software used to perform the spatial statistics calculations regarding the distribution of dengue cases. The calculations made use of the Geocentric Reference System for the Americas, 2000 edition (Sistema de Referência para as Américas, SIRGAS), from the German Geodetic Research Institute (Deutsches Geodätisches Forschungsinstitut, DGFI; Munich, Germany).

QGIS was used to determine the spatial distribution of dengue cases. This enabled construction of heat maps showing the intensities of events in the region and the evidence of occurrences, in real time.

Spearman’s test was used for correlation calculations. This is a nonparametric test that is recommended for use when it is undesirable to make any assumption of normal distribution or presence of any other variable distribution. This coefficient is based on observation points within each variable and on differences between the points observed, expressed as variables X and Y, for the same object of study.

Approximately 6,504 cases were georeferenced, out of the 7,524 cases reported, covering 86.44% of the total number of cases registered. The other 1,020 cases of dengue were not included either because of incompatibility of address with the cartographic references of the municipality or because the inclusion criteria were not met. The arithmetic “rule of three” was applied to determine the percentage increase in dengue over the last five years.

The incidence rate (IR) calculation was made by taking the number of cases notified to SINAN (NCN) divided by the number of years in the period surveyed (NYP) and the mean population over the period (MPP), and applying these in the formula: IR = (NCN/NYP/MPP) x 100,000. Three survey periods were chosen: 2010 to 2019; 2010 to 2014; and 2015 to 2019.

### Ethics

This study was submitted to and approved by the Ethics Committee of the School of Medical Sciences of Universidade de Pernambuco (UFPE) on February 21, 2018, under opinion report number 2.503.713 (CAAE: 62649816.1.0000.5192).

## RESULTS

The incidence rate for the entire period researched (2010 to 2019) was 480.27 cases per 100,000 inhabitants. A total of 6,504 dengue cases were notified and the average population over this period was 135,422 people. Between 2010 and 2014, a total of 1,069 dengue cases were recorded, among an average population over this period of 132,415, with an incidence rate of 161.46 cases per 100,000 inhabitants. In the second half of the survey, from 2015 to 2019, there was a total of 5,435 cases among an average population of 138,532, with an incidence rate of 784.65 cases per 100,000 inhabitants. This latter rate represented a 485.97% increase in dengue cases.

**Figure 1:**
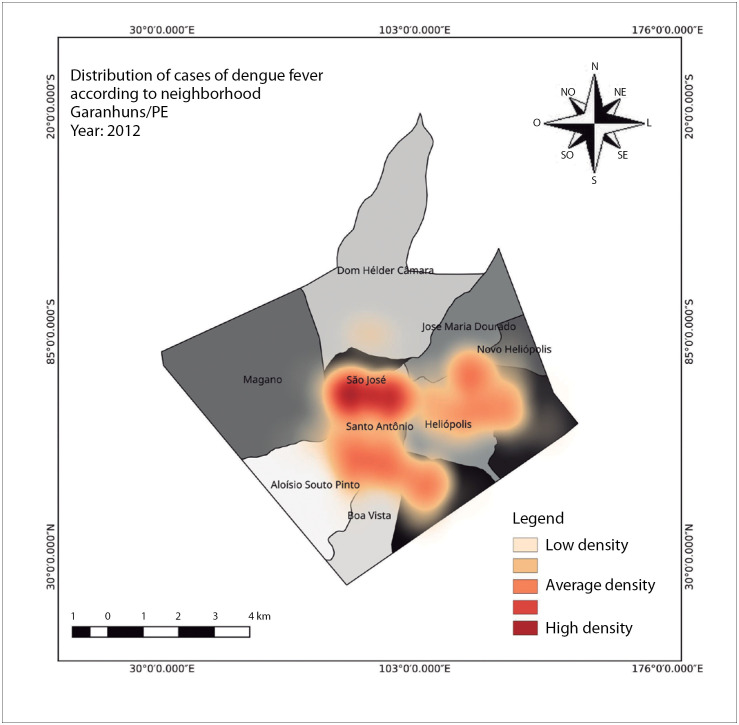
Spatial distribution of dengue cases in the municipality of Garanhuns, state of Pernambuco, in 2012, highlighting the neighborhoods of São José, Santo Antônio, Aluísio Pinto, Heliópolis, Severiano Morais and Francisco Simão, The year 2012 was the year with the highest incidence over the five-year period from 2010 to 2014.

Source: City Health Department - Garanhuns/PE.

In the first half of the period surveyed, i.e. between 2010 and 2014, 2012 was the year in which the highest incidence occurred, with 271.4 cases per 100,000 inhabitants. In that year, a total of 356 cases were notified and the average population was 131,169. Figure 1 shows the spatial distribution of dengue cases in the year 2012, in the neighborhoods of São José, Santo Antônio, Heliópolis, Severiano Morais, Aluisio Pinto, Boa Vista and Francisco Simão.

**Figure 2:**
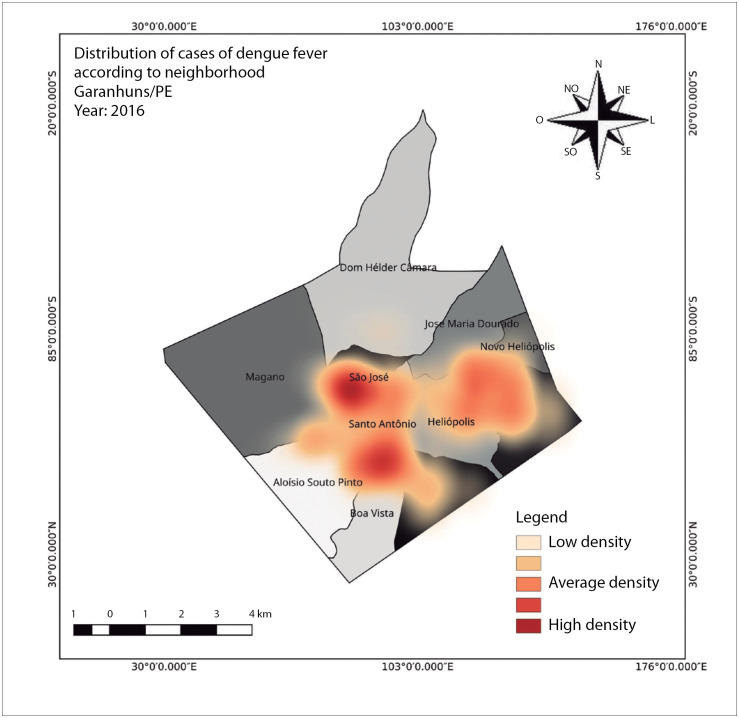
Spatial distribution of dengue cases in the municipality of Garanhuns, state of Pernambuco, in 2016, highlighting the neighborhoods of São José, Santo Antônio, Aluísio Pinto, Heliópolis, Severiano Morais and Boa Vista, The year 2016 was the year with the highest incidence over the five-year period from 2015 to 2019.

Source: City Health Department - Garanhuns/PE.

The highest occurrence of dengue cases in the period between 2015 and 2019 was in 2016. In that year, a total of 3031 cases of dengue was recorded among a population of 137,810 inhabitants. The incidence for this particular year was 2,199 cases per 100,000 inhabitants. 2016 was considered to be an epidemic year in the municipality, with greatest spatial distribution in the neighborhoods of São José, Santo Antônio, Heliópolis, Severiano Morais, and Boa Vista. The population densities in these neighborhoods were 4,797, 4,269, 3,710, 3,567 and 2,306 inhabitants per km^2^, respectively (Figure 2).

The year 2016 represented the peak of dengue momentum in the municipality, with an incidence rate of 2,199 dengue cases per 100,000 inhabitants. The rainfall was not significantly higher than normal; the average temperature was 31.4 °C and the average humidity was 31.4%. These acted as ideal conditions for proliferation of transmitting agents and influenced the rate of occurrence of dengue, can be seen in Table 1.

**Table 1:** Incidence of dengue per 100,000 inhabitants in the municipality of Garanhuns, in relation to environmental factors, over the period from 2010 to 2019

Year	Population	Average rainfall (mm)	Average temperature (°C)	Humidity %	Number of cases	Incidence (per 100,000)
2010	129,408	93.84	26.2	48.1	285	220.23
2011	130,303	79.25	25.7	50.0	354	271.67
2012	131,169	30.08	28.8	49.1	356	271.40
2013	135,138	60.34	29.6	35.6	42	31.07
2014	136,057	90.55	29.4	37.3	32	23.51
2015	136,949	49.15	30.3	34.5	1,375	1,004.00
2016	137,810	45.10	31.4	31.4	3,031	2,199.00
2017	138,642	107.29	29.2	38.8	464	334.67
2018	138,983	64.29	30.2	37.4	195	140.30
2019	139,788	69.18	30.6	38.8	370	264.68

The influence of demographic density on the incidence of dengue can be seen in Table 2. This depicts the relationship between the number of dengue cases and the density of inhabitants according to neighborhood. Annual data on average temperature, accumulated precipitation and average relative humidity were individually compared with the number of dengue cases. These correlations were not statistically significant (P > 0.0) (Table 3).

**Table 2:** Population density in relation to the number of dengue cases, according to neighborhood. The significance of associations was higher for the neighborhoods of Heliopolis, São José and Boa Vista (population densities of these neighborhoods are therefore indicated in bold]

Neighborhood	Average population	Area (km^2^)	Density of inhabitants per km^2^	Average number of cases
Severiano Morais	20,833	5.84	3,567.00	28.2
Heliópolis	20,246	5.47	**3,710.14**	178.3
Magano	12,672	15.40	822.88	89.7
Aluísio Pinto	12,406	6.88	1.803.00	65.1
São José	12,138	2.53	**4,797.00**	71.6
Francisco Simão	11,756	4.30	2,734.00	39.6
Dom Helder	4,359	15.40	283.08	21.5
Boa Vista	11,184	4.85	**2,306.00**	156.3
Santo Antônio	6,232	1.46	4,269.00	24.4
José M. Dourado	2,323	5.12	453.73	29.1
Novo Heliópolis	2,295	1.73	1,326.00	22.7
Dom Thiago	423	0.31	1,591.00	0.2

**Table 3:** Results from Spearman’s correlation test on variables in relation to the number of dengue cases per year in Garanhuns, over the years from 2010 to 2019

Variable	Correlation	P-value
Average annual temperature (°C)	0.4061	0.2474
Average annual rainfall (mm)	-0.3091	0.3871
Average annual relative humidity (%)	-0.3091	0.3871

## DISCUSSION

The incidence recorded over the 10 years of the survey, between 2010 and 2019, was 474.92 cases per 100,000 inhabitants, with a total of 6,504 cases. When we divided the survey into two periods, i.e. 2010-2014 and 2015-2019, we found the following levels of incidence per 100,000 inhabitants: In the first half of the survey, with a total of 1069 cases, the incidence was 161.46. In comparison, in the second half of the survey, a total of 5,435 cases were registered and the incidence was 784.65, thus representing an increase of 485.97% in the number of dengue cases.

Figure 1 represents 2012, the year with the highest incidence in the first period of the survey, between 2010 and 2014. There was a total of 356 cases in this year, representing an incidence rate of 271.4 cases per 100,000 inhabitants. In the second period of the survey, between 2015 and 2019, the highest incidence were observed in 2016, and this is represented in Figure 2. The incidence rate was 2,199 cases per 100,000 inhabitants, from a total of 3,031 cases recorded.

High spatial distribution in certain neighborhoods was also observed. Table 2 shows that some neighborhoods with high population densities usually had more cases of dengue than did low-density neighborhoods.

Temperature, relative humidity and precipitation did not show any associations with occurrences of dengue in the municipality. These climatic correlations were not statistically significant (P > 0.05). Some studies carried out in northeastern, southeastern and northern Brazil and abroad^16^^,^^18^^,^^19^^,^^20^ have shown correlations contrary to those of the present study, given that they reported associations between climatic factors and the incidence of dengue. On the other hand, some other studies^28^^,^^29^ have confirmed the lack of correlation of climatic factors with the incidence of dengue.

In the city of Mossoró, state of Rio Grande do Norte, a study covering the years from 2001 to 2007 found that the incidence of dengue was 47.01 cases per 10,000 inhabitants, with greater intensities between the months of February and May. The average temperature for the entire period was 27 °C and the average precipitation was 69 mm.^16^ A study on three municipalities in the state of Paraíba, northeastern Brazil, covering the years 2012 and 2013, found that *Aedes aegypti* was capable of completing its life cycle in temperatures that ranged from 22 °C to 36 °C.^17^

A study carried out in Fortaleza covering the years 2001 to 2013 found that the climatic dynamics of the disease showed a significant variation of precipitation and humidity, with temperatures ranging between 25 °C and 28.8 °C.^18^ In São Luis, state of Maranhão, a study covering 2002 to 2012 that included meteorological variables showed that the incidence of dengue fluctuated according to climatic periods, such that greater numbers of dengue cases occurred during the rainy season and at times of higher temperatures.^19^ Another study carried out in northeastern Brazil in Maceió, state of Alagoas, covering 2010 to 2016, evaluated the correlation of meteorological parameters with the incidence of dengue. It showed that precipitation and humidity influenced the epidemiology of dengue.^20^

A study carried out in Pakistan showed that the incidence of the disease was influenced by climatic factors, such that the transmission rates among mosquitoes were higher within a favorable temperature range from 28 °C to 32 °C.^21^ Laboratory data on larval development of Aedes aegypti have confirmed that these temperatures favor multiplication of these larvae, consequently enabling greater production of vectors and producing increased incidence of dengue.^22^

In an urban area of the city of São Paulo, Brazil, a study showed that the oviposition rates of *Aedes aegypti* and *Aedes albopictus* were influenced by the maximum and minimum temperatures.^23^

Temperature influences mosquitos’ life cycles and plays a crucial role in the incidence of dengue. Analyzing the effects of temperature variations in cities can lead to preventive identification of thermal comfort zones favorable to the survival of mosquito populations.^24^ Knowing how environmental conditions influence the dynamics of dengue epidemics is important for responding to its epidemics and for predicting the geographical and seasonal spread of the disease.^25^

Although there is no statistical association between temperature and dengue cases, it appears that dengue peaks coincide with temperature spikes.^26^ This hypothesis was reinforced through a wintertime study carried out in Taiwan, which is a in subtropical region, where a low temperature of 13.8 °C resulted in the near disappearance of *Aedes aegypti*.^27^ A study on the impact of dengue in the state of Tocantins, Brazil, revealed that climatic conditions did not influence proliferation of dengue but, rather, the conditions that would be ideal for reproduction of the vector.^28^ An ecological study carried out in Araguaína, Tocantins, also did not find any correlation with climatic variables and concluded that these variables contributed to vector proliferation, but did not influence the spread of dengue.^29^

Data from 13 weather stations in Delhi, India, over the period from 2006 to 2015, indicated that there was a strong association between the incidence of dengue and the temperature, humidity, wind speed, summertime, settlement density and vegetation.^30^ In China, results from sensitivity analyses indicated that temperature can be an effective or facilitating barrier for vector-borne diseases and can result in complex disease control.^31^ Variations in daytime temperature, precipitation and relative humidity have had statistically significant results in multiple linear regressions for the number of dengue cases.^32^ The association between climatic factors and dengue incidence suggests that application of any prospective dengue early warning system should be done on a local or regional basis rather than on a national scale.^33^

The years of 2015 and 2016 were years of drought for the city of Garanhuns, and this may have favored an increase in the number of mosquito breeding sites and thus may have caused the dengue epidemic of 2016. On the other hand, in some cases of dengue. its incidence may have been related to the Zika virus, as reported in a cross-sectional study carried out in the state of Pernambuco, which pointed out differential diagnoses of arboviruses, carried out between January and April 2015, based on clinical and epidemiological criteria. Among these diagnoses, 1,046 suspected cases were recorded, of which 895 (86%) were classified as probable cases of the Zika virus and 151 (14%) as cases of dengue.^34^ However, of 8,429,735 cases of arboviruses reported in Brazil in 2015 and 2016, only 5% were suspected of being caused by the Zika virus.^35^

The limitations of this study comprised its inclusion criteria, i.e. the subjects needed to have an address in the municipality of Garanhuns, their cases needed to have been notified to SINAN and a diagnosis of dengue needed to have been made. Patients who did not meet these inclusion criteria and those whose addresses could not be georeferenced due to lack of information were excluded: these exclusions corresponded to 13.78% of the total number of notified cases.

## CONCLUSION

The climate and local geography of the study area, characterized by wide variations in temperature and precipitation, with prolonged periods of drought and densely populated neighborhoods, may have contributed to greater reproduction and dissemination of the transmitting vector. This may have led to differences in dengue incidence rates over the last five years, thereby increasing the number of outbreaks and even epidemics.

These results should serve as the basis for the creation of new control and continued prevention strategies. They also demonstrate that there is a need for greater in-depth study of the spatial distribution of dengue, using regression analysis.
